# Deep Learning for the Preoperative Diagnosis of Metastatic Cervical Lymph Nodes on Contrast-Enhanced Computed ToMography in Patients with Oral Squamous Cell Carcinoma

**DOI:** 10.3390/cancers13040600

**Published:** 2021-02-03

**Authors:** Hayato Tomita, Tsuneo Yamashiro, Joichi Heianna, Toshiyuki Nakasone, Tatsuaki Kobayashi, Sono Mishiro, Daisuke Hirahara, Eichi Takaya, Hidefumi Mimura, Sadayuki Murayama, Yasuyuki Kobayashi

**Affiliations:** 1 Department of Radiology, St. Marianna University School of Medicine, 2-16-1 Sugao, Miyamae-ku, Kawasaki, Kanagawa 216-8511, Japan; mimura@marianna-u.ac.jp; 2 Department of Radiology, Graduate School of Medical Science, University of the Ryukyus, 207 Uehara, Nishihara, Okinawa 903-0215, Japan; clatsune@yahoo.co.jp (T.Y.); anna1150@med.u-ryukyu.ac.jp (J.H.); sadayuki@med.u-ryukyu.ac.jp (S.M.); 3 Department of Oral and Maxillofacial Surgery, Graduate School of Medical Science, University of the Ryukyus, 207 Uehara, Nishihara, Okinawa 903-0215, Japan; nakasone4266@gmail.com; 4 Department of Advanced Biomedical Imaging Informatics, St. Marianna University School of Medicine, 2-16-1 Sugao, Miyamae-ku, Kawasaki, Kanagawa 216-8511, Japan; t_kobayashi@vis-ionary.com (T.K.); yasukoba2@gmail.com (Y.K.); 5 Department of AI Research Lab, Harada Academy, 2-54-4 Higashitaniyama, Kagoshima, Kagoshima 891-0113, Japan; mishiro@harada-gakuen.ac.jp (S.M.); rt.hirahara@harada-gakuen.ac.jp (D.H.); 6 School of Science for Open and Environmental Systems, Graduate School of Science and Technology, Keio University, 3-14-1 Hiyoshi, Kohoku-ku, Yokohama, Kanagawa 223-8522, Japan; etakaya@keio.jp

**Keywords:** deep learning, cervical lymph node, convolutional neural network, level, squamous cell carcinoma

## Abstract

**Simple Summary:**

Cervical lymph node (LN) metastasis in patients with oral squamous cell carcinoma is one of the important prognostic factors. Pretreatment cervical nodal staging is performed using computed tomography (CT) as the first-line examination. However, imaging findings focused on morphology are not specific for detecting cervical LN metastasis. In this study, deep learning (DL) analysis of pretreatment contrast-enhanced CT was evaluated and compared with radiologists’ assessments at levels I–II, I, and II using the independent test set. The DL model achieved higher diagnostic performance in discriminating between benign and metastatic cervical LNs at levels I–II, I, and II. Significant difference in the area under the curves of the DL model and the radiologists’ assessments at levels I–II and II were observed. Our findings suggest that this approach can provide additional value to treatment strategies.

**Abstract:**

We investigated the value of deep learning (DL) in differentiating between benign and metastatic cervical lymph nodes (LNs) using pretreatment contrast-enhanced computed tomography (CT). This retrospective study analyzed 86 metastatic and 234 benign (non-metastatic) cervical LNs at levels I–V in 39 patients with oral squamous cell carcinoma (OSCC) who underwent preoperative CT and neck dissection. LNs were randomly divided into training (70%), validation (10%), and test (20%) sets. For the validation and test sets, cervical LNs at levels I–II were evaluated. Convolutional neural network analysis was performed using Xception architecture. Two radiologists evaluated the possibility of metastasis to cervical LNs using a 4-point scale. The area under the curve of the DL model and the radiologists’ assessments were calculated and compared at levels I–II, I, and II. In the test set, the area under the curves at levels I–II (0.898) and II (0.967) were significantly higher than those of each reader (both, *p* < 0.05). DL analysis of pretreatment contrast-enhanced CT can help classify cervical LNs in patients with OSCC with better diagnostic performance than radiologists’ assessments alone. DL may be a valuable diagnostic tool for differentiating between benign and metastatic cervical LNs.

## 1. Introduction

Metastasis to the cervical lymph nodes (LNs) is one of the poor prognostic factors in patients with oral squamous cell carcinoma (OSCC). Evaluating whether the cervical LNs are benign or metastatic depends on the treatment strategy. Among patients with clinically negative LNs, 15–20% are at risk of occult LN metastasis [1]. Unnecessary surgical LN dissection without metastatic cervical LNs can lead to increased complications, while delayed dissection of LN metastases can result in disease progression. Ultrasonography (US), computed tomography (CT), magnetic resonance imaging (MRI), and fluorine-18-2-fluoro-2-deoxy-D-glucose positron emission tomography (18F-FDG PET) have been widely used for evaluating the cervical LN status in head and neck cancer patients [2,3,4]. However, the subjective nature of the morphologic criteria for visually confirming metastatic LNs on US, CT, and MRI results in diminished reproducibility and objectivity. Although several studies have recently described the usefulness of dual-energy CT to evaluate the cervical LN status in head and neck cancer patients, it is not widely used [5,6]. 18F-FDG PET has been known to be the best modality for evaluating cervical LN metastasis in these patients. However, the diagnosis of small cervical LNs for evaluating the nodal status using 18F-FDG PET is limited, owing to false-negative findings [7,8]. Additionally, the sensitivity of sentinel LN biopsy and sentinel LN imaging techniques using CT or MR lymphography and PET lymphoscintigraphy is 56–91% [9,10]. Unfortunately, metastatic cervical LNs are not easily detected on a pretreatment clinical examination. Therefore, the development of accurate diagnostic methods is required.

With the continued development of artificial intelligence, deep learning (DL) has been applied to medical imaging for tissue characterization, outcome prediction, and automated detection [11,12,13,14,15]. DL enables the parameters to increase and handle complex tasks by increasing the layers of the neural networks that imitate models of brain structures connecting a large number of neurons. Convolutional neural network (CNN), one of the DL architectures, consists of convolutional and pooling layers. Convolutional layers convert some pixels in the grid into one pixel and extract the image features called a feature map. Pooling layers decrease the amount of calculation and adapt to the misalignment of images by reducing the data of the feature map. CNNs can play an important role in interpreting medical imaging without subjective assessment. Previous studies have shown how CNN could effectively assess the malignancy of hepatocellular carcinoma and prostate cancer lesions [13,14]. Furthermore, DL was able to help discriminate between benign and metastatic cervical LNs in patients with OSCC [16]. However, its value based on the American Head and Neck Society cervical regional lymph node level system, which has been used to determine the extent of LN dissection and indication for radiotherapy, has not been evaluated. Although LNs at levels I and II are known to drain from the lymphatic tract of the OSCC, identifying metastatic cervical LNs remains challenging because of oral and sinonasal inflammation or insufficient malignant deposits. In addition, prophylactic cervical neck dissection is frequently performed level-by-level in clinical practice since cervical LNs metastasis in OSCC can occur even if these are clinically diagnosed as benign lesions. Hence, unnecessary neck dissection can be prevented if the benign or metastatic LNs can be distinguished for each level. Therefore, we aimed to clarify the diagnostic performance of DL in differentiating between benign and metastatic level I–II, I, and II cervical LNs on contrast-enhanced CT in patients with OSCC.

## 2. Results

Patient characteristics are summarized in Table 1. Table 2 shows the number of LNs at each level in each set.

### 2.1. Diagnostic Performance of the Deep Learning Model in the Validation and Test Sets

In the validation set, the DL model achieved a diagnostic accuracy rate/area under the receiver operating characteristic curve (AUC) of 97.5%/0.964 at levels I–II. A summary of the diagnostic performances of the DL model and the radiologists’ assessments in the test set is shown in Table 3. The DL model achieved a diagnostic accuracy rate/AUC of 85.9%/0.898 at levels I–II, 83.9%/0.824 at level I, and 90.9%/0.967 at level II.

### 2.2. Diagnostic Performance of the Readers in the Test Set

**Figure 1:**
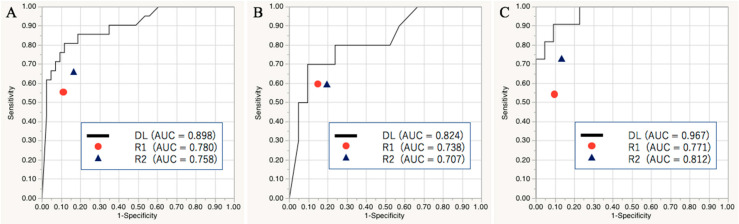
Areas under the receiver operating characteristic curves showing the deep learning model and radiologists’ ability to identify metastatic cervical lymph nodes at levels I–II (**A**), I (**B**), and II (**C**) in the test set.

**Figure 2:**
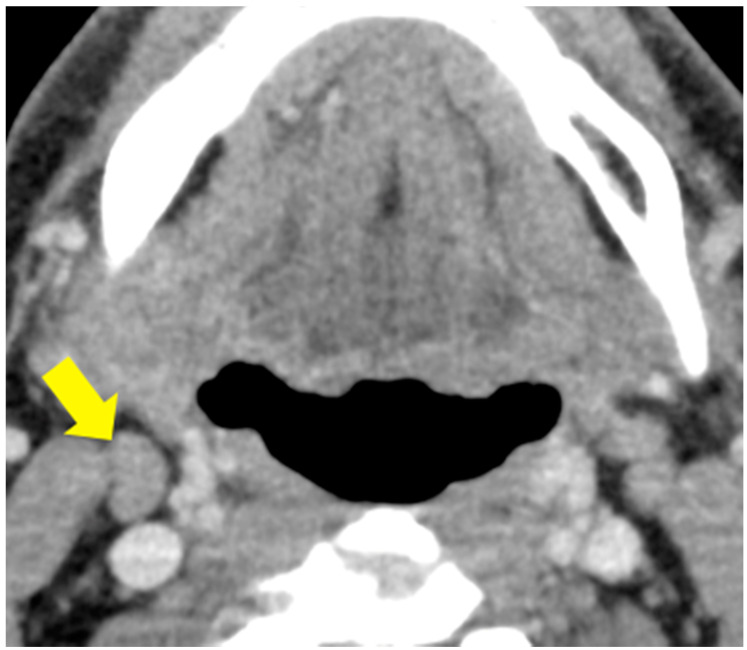
A representative case of cervical LN at level ⅡA on the right in a 56-year-old man with gingival cancer of T4 classification whose diagnosis differed between the deep learning (DL) model and radiologists’ assessments. The targeted LN size was 8.2 mm. The DL model diagnosed it as a metastatic LN, while two radiologists diagnosed it as a benign LN.

Figure 1 shows the receiver operating characteristic curves of the DL model and the radiologists’ assessments. Significant differences in the AUCs at levels I–II (0.898 [DL] vs. 0.780 [R1] and 0.758 [R2]; both, *p* < 0.05) and level II (0.967 [DL] vs. 0.771 [R1] and 0.812 [R2]; both, *p* < 0.05) between the DL model and the radiologists’ assessments were found. The DL model was more accurate at levels I–II (85.9% [DL] vs. 78.1% [R1] and 78.1% [R2]), level I (80.6% [DL] vs. 77.4% [R1] and 74.2% [R2]), and level II (90.9% [DL] vs. 78.8% [R1] and 81.4% [R2]). The DL model improved 16 diagnostic decisions of the readers. For the benign LNs at levels I–II, the DL model accurately diagnosed four and seven LNs that were misdiagnosed by R1 and R2, respectively, while one and two LNs that were accurately diagnosed by the readers were not accurately diagnosed by the DL model. For the metastatic LNs, the DL model improved four and one LNs that were diagnosed as benign lesions by R1 and R2, respectively, while two and one LNs that were accurately diagnosed by the readers were not accurately diagnosed by the DL model. A representative case of different diagnostic decisions between the DL model and radiologists is shown in Figure 2.

## 3. Discussion

In this study, the DL model achieved higher diagnostic performance in discriminating between benign and malignant cervical LNs on contrast-enhanced CT in patients with OSCC. In the test set, a significant difference in the AUCs of the DL models and radiologists was observed. Our results suggest that preoperative cervical nodal status at level I and II in patients with OSCC can be evaluated by DL.

The following CT and MR morphologic criteria have been widely used to determine the malignancy of cervical LNs in patients with head and neck cancer: Nodal size, peripheral shape, heterogeneous enhancement, and clustering of LNs. The diagnosis of LNs has depended on the judgment of radiologists and clinicians. Park et al. have reported that the sensitivity/specificity/accuracy of CT/MR for the visual assessment of cervical LNs in patients with head and neck SCC were 42/94/85% and 70/91/84 % at the bilateral levels I and II, respectively [2].

Kann et al. [17] demonstrated that a test set evaluated using DualNet DL achieved a sensitivity/specificity/accuracy of 84/87/86%, respectively. Similarly, an AUC of 0.91 for the assessment of the overall cervical LNs in head and neck cancer patients was found. However, no previous studies have reported the diagnostic performance of DL models at each LN level in OSCC patients. Our study can provide useful information about preoperative evaluation of cervical LN at levels Ⅰ and Ⅱ. In two studies, the entire LN was segmented for the assessment of the cervical LN status using CNNs [17,18]. However, in our study, the largest slice of the cervical LNs was used to simplify the workflow and avoid unnecessary CNN calculations. The center of the cervical LN can play a key role in its evaluation using CNNs. Ariji et al. [16] have described that DL with AlexNet could be useful in distinguishing benign and metastatic LNs from overall cervical LNs in OSCC patients. However, no significant difference between the DL model and the radiologists’ assessments was found. Segmented CT images using an arbitrary-sized square included soft tissue structures around LNs in their study. Meanwhile, segmentation of the border of LNs without soft tissue structures was performed in our study. That might lead to improvement of the diagnostic performance using the DL model. However, these approaches of segmentation are not entirely automated and require human intervention. Fully automatic detection and classification of cervical LNs are required to improve the reproducibility. The targeted area of the cervical LN dissection should be precisely determined to minimize complications and the risk of residual tumor. For level-based analysis, especially at level I, small deposits of cancer cells that may not influence the appearance of the LN’s internal architecture on CT can lead to false negatives. Thus, while CT exhibits high specificity for metastatic LN, it is not particularly sensitive. In our study, the accuracy of DL assessment of cervical LNs was superior to those of visual assessments. CNNs learn by reducing the differences between input and output data using backpropagation and loss function and identifying the useful connections within the neural network by itself. A large amount of excellent quality input data would allow high CNN performance. We utilized transfer learning using Xception in this study. In transfer leaning, the CNN architecture is pretrained from a large dataset, such as ImageNet, as the imaging features have already been extracted. Therefore, transfer learning improves the model’s performance in limited datasets, and previous studies have utilized this approach for medical imaging [19,20,21]. Regarding showing a higher diagnostic performance of CNN compared with radiologists, CNN may have extracted some sort of image features that the radiologists could not recognize, which contributed to the discrimination between benign and metastatic cervical LNs.

There were several limitations to this study. First, selection bias was present, because patients who were suspected of having metastatic cervical LNs underwent dissection. Second, only a small number of LNs were used to create the DL model in this retrospective study. The cervical LNs at levels I and II were evaluated in the validation and test sets while LNs at levels I to V were included in the training set. Third, the image preprocessing protocol and DL model algorithm that we adopted might not be optimally suited for discriminating between benign and metastatic LNs since DL models for medical imaging are not yet sufficiently developed. Data volume and quality have a key role in improving the performance of DL models. Additionally, CT images that were acquired using two CT scanners were used. Although image standardization was performed, different image intensities originating from two scanners can affect the consistency of our results. For future studies, using the same CT scanner and protocol are preferable. Fourth, the diagnostic values of DL models have not been compared with those of PET-CT, which has widely spread as the best modality for the assessment of cervical LNs in head and neck cancers. The comparison leads to confirmation of the clinical significance of the DL models. Therefore, further large, multicenter studies are required to investigate the DL model with the optimal protocols for each level, compared with PET-CT. Fifth, there were seven patients who underwent dissection of their cervical LNs after the primary surgery. Postoperative inflammation might influence the LNs since cross sectional imaging for the assessment of recurrence is recommended after 2 to 3 months to avoid false lesions [22]. Sixth, eight cervical LN metastases were not identified on CT due to rapid growth. Hence, shortening the time between the CT examination and surgery is needed.

## 4. Materials and Methods

### 4.1. Ethical Statement

This retrospective study was approved by the Bioethics Committee of St. Marianna University School of Medicine (ethical code: 4469); the committee waived the requirement for informed consent due to the design of the study. All procedures were conducted according to the Declaration of Helsinki.

### 4.2. Subjects

**Figure 3:**
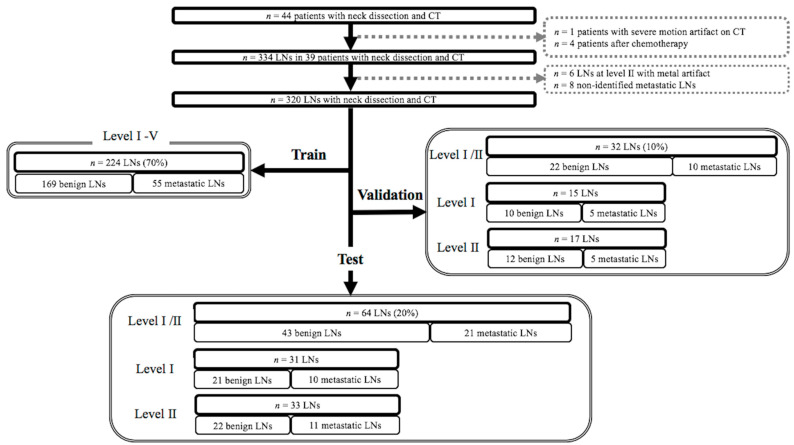
Flowchart of cervical lymph nodes included in the training, validation, and test sets.

The study flowchart is shown in Figure 3. We reviewed our electronic medical records to identify patients with OSCC who underwent neck LN dissection and contrast-enhanced CT within 1 month before neck dissection between April 2013 and November 2017. The inclusion criteria were as follows: (1) Histopathologically confirmed OSCC (tongue cancer, gingival cancer, and floor of the mouth cancer); (2) histopathologically confirmed benign and metastatic cervical LNs at levels I–V; and (3) available preoperative CT data. The exclusion criteria were motion artifacts on CT (*n* = 1), preoperative chemotherapy (*n* = 2), and induction chemotherapy (*n* = 2). In total, 39 patients were enrolled in this study. The mean interval between cervical neck dissection and CT was 21.3 ± 8.9 days. Among 39 patients, 31 underwent primary resection and neck dissection and 7 underwent cervical neck dissection after primary resection based on the suspicion of metastatic cervical LNs. For the seven patients, the median interval between initial surgery and CT was 181 (range, 44–308) days.

### 4.3. Computed Tomography

CT from the base of the skull to the bottom of the neck was performed using 320-row scanners (Aquilion ONE; Canon Medical Systems, Otawara, Tochigi, Japan) for 23 patients and 64-row scanners (LightSpeed VCT; GE Healthcare, Milwaukee, WI, USA) for 16 patients according to the following protocols: For 320-row CT scanners: Collimation, 320 × 0.5 mm; tube voltage, 120 kVp; tube current, automatic exposure control; gantry rotation time, 0.5 s; and beam pitch, 0.813. For 64-row CT scanners: Collimation, 64 × 0.5 mm; tube voltage, 120 kVp; tube current, automatic exposure control; gantry rotation time, 0.4 s; and beam pitch, 0.984. CT images with a 2-mm slice thickness without any overlap of serial sections were used. The imaging field of view was 230 × 230 mm. Iodine contrast material of 100 mL (300 mg I/mL) was intravenously injected at 1.5 mL/s for both protocols.

### 4.4. Labeling of Cervical Lymph Nodes and Targeted Lymph Node

Twenty-five patients underwent bilateral radical neck dissection, 11 underwent unilateral radical neck dissection, and 3 underwent unilateral supraomohyoid neck dissection. During surgery, the surgeon identified the cervical LNs for dissection using preoperative CT images. The operators set aside cervical LNs to determine their relative positions with reference to the size and location of LNs, vessels, muscles, salivary glands, and bones on these images. The dissected cervical LNs were stained with hematoxylin and eosin and evaluated by pathologists. LNs with histopathologically proven metastasis were labeled one-by-one at each level (levels I–IV). Initially, 334 cervical LNs were identified. However, six LNs were excluded because of severe metallic artifacts on CT images. Eight metastatic LNs were also excluded because they were not detected on CT owing to their rapid enlargement after performing CT. Therefore, 320 cervical LNs, comprising 234 benign and 86 metastatic LNs, at levels I–V were included in this study. We randomly categorized the cervical LNs into three sets: A training set at levels I–V (n = 224 [70%], 169 benign and 55 metastatic), a validation set at levels I–II (n = 32 [10%], 22 benign and 10 metastatic), and a test set at levels I–II (n = 64 [20%], 43 benign and 21 metastatic). In the validation and test sets, cervical LNs at levels III–V were not used because the necessary sample sizes for each level, as mentioned in the “statistical analysis” section, were unavailable, which could weaken the statistical power.

### 4.5. Image Preprocessing for Deep Learning

**Figure 4:**
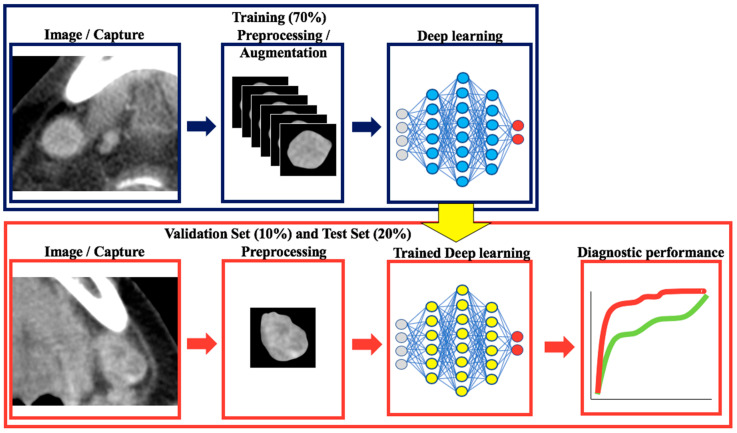
Workflow of this study.

The study workflow is shown in Figure 4. Three CT images, namely the image showing the largest cross-sectional area of the targeted LN and the adjacent images (one cranial and one caudal image), were obtained using OsirixMD software (Pixmeo, Bernex, Switzerland). The margin of the LNs on the selected images were contoured as close as possible by a single radiologist (**blinded** with 9 years of experience).

All images were resized to 300 × 300 pixels. All images were normalized and divided by 255 before the augmentation. The resized images were augmented by horizontal flip, vertical flip, width shift, and height shift. The programming language used for augmentation was Python 3.6 (https://www.python.org).

### 4.6. Classification with Convolutional Neural Networks and Transfer Learning

**Figure A1:**
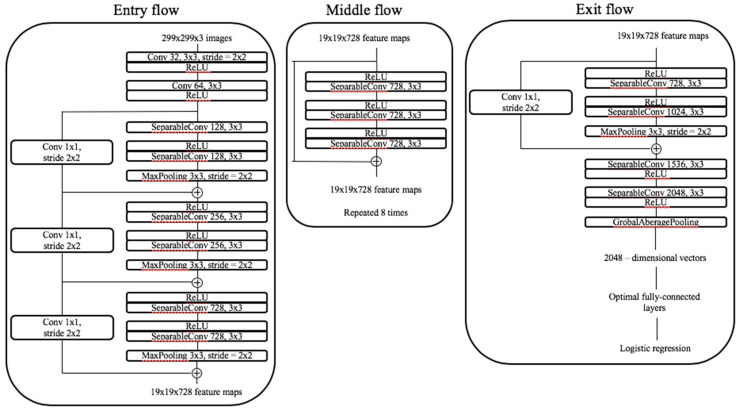
The architecture of Xception model.

In this study, the network architecture was based on the Xception architecture [23]. This network comprised three flows, namely entry flow, middle flow, and exit flow. Each flow is composed of several modules called Inception, which is a component of GoogleNet [24]. A detailed description of the Xception architecture is given in Appendix A (Figure A1). For our experiment, we used the Xception architecture pretrained on the ImageNet dataset. Only the Exit flow of the network was fine tuned to our dataset to classify benign and metastatic cervical LNs. Early stopping was conducted to avoid overfitting in the training set. This method stops training without fixing the number of epochs when validation loss is confirmed. For the validation and test sets, the performance of the trained DL model was evaluated. In the test set, to match the DL model and visual assessment findings, the largest slice of the cervical LN was used for the final analysis.

### 4.7. Visual Analysis

The interpretation of CT images was based on visual assessment by two board-certified radiologists (R1 and R2, with 9 and 19 years of experience reading head and neck CT, respectively) who were blinded to patients’ clinical information, including histopathological results. Both radiologists evaluated the cervical LNs and graded them using a 4-point scale: 1 = definitely benign; 2 = likely benign; 3 = likely metastatic; and 4 = definitely metastatic. The following CT characteristics were considered to judge the scale: Shortest maximum diameter of more than 11 mm in the jugulo-digastric area and 10 mm in other cervical areas, heterogeneous enhancement or central necrosis, or loss of fatty hilum [2,3].

### 4.8. Statistical Analysis

The necessary number of LNs was calculated to evaluate the area under the curve (AUC) with a type I error of 5% and power of 80% using the R statistical package (version 3.6.1; R Project for Statistical Computing, R Foundation, Vienna, Austria). A previous study had reported an AUC of 0.801 in quantitative detection of metastatic cervical LNs in patients with OSCC [25]. Our training cohort showed a benign to metastatic LN ratio of 3:1. We estimated that a sample size of at least 27 was required.

Statistical analysis was performed using Python 3.6 or JMP pro 14.2.0 software (SAS Institute, Cary, NC, USA). In the test set, sensitivities, specificities, diagnostic accuracy rates, and AUCs of the DL model and the radiologists’ assessments were analyzed to determine their ability to differentiate between benign and metastatic cervical LNs at levels I–II, I, and II. The AUCs were compared between the DL model and the radiologists’ assessments. *p*-values <0.05 were considered to indicate a statistically significant difference.

## 5. Conclusions

In conclusion, DL can differentiate between benign and metastatic cervical LNs on preoperative contrast-enhanced CT of patients with OSCC, which can help guide treatment decisions on neck dissection in a reproducible manner. Further investigation will be required to establish the optimal diagnostic method for cervical LN status.

## Figures and Tables

**Table 1:** Patient-based characteristics.

Characteristics		Patient-Based
		*n* = 39
**Age mean ± SD**		64.0 ± 14.0
**Gender male/female**		23/16
**Primary tumor sites**	Oral tongue	26
	Gingiva	8
	Floor of mouse	5
**T stage**	T1	7
	T2	17
	T3	7
	T4	8
**N stage**	N0	7
	N1	11
	N2a	0
	N2b	13
	N2c	6
	N3a	0
	N3b	2
**Level** **Ⅰ**		121
**Ipsilateral Level** **Ⅰ**		84
**Level** **Ⅱ**		132
**Ipsilateral Level** **Ⅱ**		72
**Level** **Ⅲ**		37
**Ipsilateral Level** **Ⅲ**		19
**Level** **Ⅳ**		4
**Ipsilateral Level** **Ⅳ**		4
**Level** **Ⅴ**		26
**Ipsilateral Level** **Ⅴ**		13

**Table 2:** The number of lymph nodes (LN) at each level in train set, validation set, and test set.

Level	LN-Based					
	Train Cohort (*n* = 224)		Validation Cohort (*n* = 32)		Test Cohort (*n* = 64)	
	Benign (*n* = 169)	Metastasis (*n* = 55)	Benign (*n* = 22)	Metastasis (*n* = 10)	Benign (*n* = 43)	Metastasis (*n* = 21)
**Level** **I**	51	24	10	5	21	10
**Ipsilateral Level** **I**	29	20	7	5	15	8
**Level** **II**	64	18	12	5	22	11
**Ipsilateral Level** **II**	26	14	4	5	12	11
**Level** **III**	27	10	-	-	-	-
**Ipsilateral Level** **III**	12	7	-	-	-	-
**Level** **IV**	1	3	-	-	-	-
**Ipsilateral Level** **IV**	0	3	-	-	-	-
**Level** **V**	26	0	-	-	-	-
**Ipsilateral Level** **V**	13	0	-	-	-	-

**Table 3:** Comparisons of area under the curve (AUC), diagnostic accuracy rate, sensitivity, and specificity between deep learning models and radiologists at each level.

	AUC [95% Confidence Interval]	Accuracy	Sensitivity	Specificity
Level I/II				
Deep learning	0.898 [0.778, 0.956]	85.9	66.7	95.4
Reader 1	0.780 [0.559, 0.864] *	78.1	57.1	88.4
Reader 2	0.758 [0.587, 0.873] *	78.1	66.7	83.7
Level I				
Deep learning	0.824 [0.600, 0.936]	80.6	60.0	90.5
Reader 1	0.738 [0.497, 0.889]	77.4	60.0	85.7
Reader 2	0.707 [0.443, 0.880]	74.2	60.0	81.0
Level II				
Deep learning	0.967 [0.854, 0.993]	90.9	72.7	100.0
Reader 1	0.771 [0.546, 0.904] *	78.8	54.6	90.9
Reader 2	0.812 [0.574, 0.933] *	81.8	72.7	86.4

* Indicates a significant difference between deep learning and radiologists (*p* < 0.05).

## Data Availability

Most of the data in this study are available in this article. Some data including CT imagings are not publicly available due to data protection regulations.
